# Association Between Triglyceride‐Glucose‐Body Mass Index and Depression in Elderly Diabetic Individuals: A Population‐Based Study

**DOI:** 10.1155/ije/3433631

**Published:** 2026-02-10

**Authors:** Hong Qian, Shanglin Song, Zhengyu Zhang, Yuan Yuan, Dean Wu

**Affiliations:** ^1^ Department of Medical Administration, The First Hospital of Lanzhou University, Lanzhou, 730000, China, lzu.edu.cn; ^2^ Medical Records Department, The First Affiliated Hospital, College of Medicine, Zhejiang University, Hangzhou, 310003, China, zju.edu.cn; ^3^ Medical Department, Women and Children’s Hospital of Chongqing Medical University, Chongqing, 401147, China; ^4^ Department of Respiratory and Critical Care Medicine, The Third people’s Hospital of Gansu Province, Lanzhou, 730000, China

**Keywords:** depression, elderly diabetes, insulin resistance, NHANES, TyG-BMI

## Abstract

**Background:**

Although the association between insulin resistance (IR) and depression has been extensively studied, the relationship between triglyceride‐glucose‐body mass index (TyG‐BMI) and depressive symptoms in elderly patients with diabetes remains unclear. This study aimed to investigate the association between TyG‐BMI and depression risk in older adults with diabetes using data from the National Health and Nutrition Examination Survey (NHANES).

**Methods:**

This retrospective cross‐sectional study included a total of 2584 participants aged ≥ s65 years with diabetes from NHANES (2005–2018). Depression was assessed using the Patient Health Questionnaire‐9 (PHQ‐9), with a score ≥ 10 defining clinically significant depression. Multivariable logistic regression and restricted cubic spline models were employed to analyze the TyG‐BMI–depression association, adjusting for sociodemographic, lifestyle, and disease‐related covariates. In addition, subgroup analyses were also conducted.

**Results:**

Participants in the highest TyG‐BMI quartile (Q4: > 314.04) exhibited a significantly higher risk of depression compared to the lowest quartile (Q1: < 232.44), with an adjusted odds ratio (OR) of 2.972 (95% CI: 1.264–6.988). A dose‐response analysis revealed a linear positive relationship between TyG‐BMI and depression risk (*p* for nonlinearity = 0.066). Subgroup analyses showed no significant interactions by gender, education level, or other stratification factors (all *p* > 0.05).

**Conclusion:**

Elevated TyG‐BMI is independently associated with increased depression risk in elderly diabetic patients, highlighting its potential as a biomarker for metabolic‐psychiatric comorbidity. Further studies are warranted to validate TyG‐BMI’s utility in early depression screening and intervention strategies.

## 1. Introduction

Depression is one of the leading causes of disability worldwide [1]. According to a Global Burden of Disease (GBD) study, approximately 350 million people worldwide are affected by depression, with the prevalence rate among older adults increasing significantly with age [2]. Depression not only diminishes the quality of life for those affected but is also associated with an increased risk of chronic conditions such as cardiovascular disease (CVD) and diabetes, placing a substantial economic burden on society [3, 4]. Research indicates that early screening and intervention for depression can effectively slow disease progression and reduce the risks of suicide and comorbid mortality [5]. Therefore, identifying biomarkers and developing risk prediction tools for depression is of significant importance to public health [6, 7].

Insulin resistance (IR), a core mechanism of metabolic syndrome, exhibits a bidirectional relationship with depression [8–10]. Chronic inflammation, oxidative stress, and dysregulation of the hypothalamic‐pituitary‐adrenal (HPA) axis induced by IR disrupt neurotransmitter synthesis (e.g., serotonin), exacerbating depressive symptoms [11]. Concurrently, depression is often accompanied by glucose and lipid metabolism abnormalities, forming a “metabolic‐psychological” vicious cycle [12]. Clinical studies demonstrate significant positive correlations between IR‐related indices (e.g., HOMA‐IR) and depression scores, suggesting metabolic dysregulation plays a pivotal role in depression pathogenesis [13].

Depressed patients frequently exhibit impaired glucose metabolism, elevated BMI, and visceral adiposity, with obesity exacerbating IR through adipokine dysregulation [14, 15]. Traditional IR assessments (e.g., HOMA‐IR) rely on insulin measurements, limiting clinical utility. In contrast, the triglyceride‐glucose‐body mass index (TyG‐BMI), integrating fasting triglycerides, glucose, and BMI, provides a comprehensive evaluation of insulin sensitivity and obesity‐related metabolic risks [16, 17]. Evidence indicates that TyG‐BMI outperforms single metrics in predicting type 2 diabetes and CVD, with its association with depressive symptoms being particularly pronounced in diabetic populations [18, 19].

Although the association between IR and depression has been well established, the relationship between TyG‐BMI and depressive symptoms in elderly patients with diabetes remains unclear. To address this knowledge gap, this study utilized data from the National Health and Nutrition Examination Survey (NHANES) to systematically investigate the potential association between TyG‐BMI and depression risk among older adults with diabetes.

## 2. Methods

### 2.1. Study Design and Population

This retrospective cross‐sectional study used data from the NHANES. NHANES collects health and nutrition information from U.S. residents using a step‐by‐step sampling method. We analyzed data from seven survey periods (2005–2018). Given that the NHANES dataset comprises anonymized information accessible to the public and has already obtained ethical clearance from the National Center for Health Statistics Institutional Review Board (IRB), further IRB authorization was deemed unnecessary for this particular secondary analysis. Detailed methods and raw data can be downloaded from the official website (https://wwwn.cdc.gov/nchs/nhanes).

**FIGURE 1:**
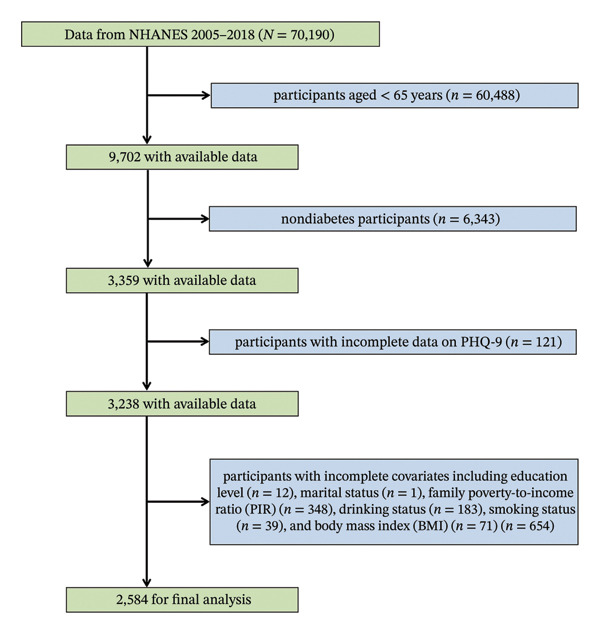
Flowchart of the sample selection from the 2005–2018 NHANES.

From the 2005–2018 NHANES, a total of 70,190 participants were initially identified. The selection process involved several exclusion criteria: individuals under 65 years of age were excluded (*n* = 60,488). Participants who were not diagnosed with diabetes were excluded (*n* = 6343). Participants with incomplete responses to the Patient Health Questionnaire‐9 (PHQ‐9) (*n* = 121) were also excluded. Further exclusions were made for incomplete data on various covariates, such as education level (*n* = 12), marital status (*n* = 1), family poverty‐to‐income ratio (PIR) (*n* = 348), drinking status (*n* = 183), smoking status (*n* = 39), and BMI (*n* = 71), amounting to a total of 654 exclusions. Following these exclusions, the final analysis included 2584 eligible participants (Figure 1).

### 2.2. Assessment of Diabetes

The diagnosis of diabetes was made in accordance with the criteria set forth by the American Diabetes Association (ADA) Standards and also took into account the data from self‐reported questionnaires. Individuals were deemed to have diabetes if they met any one of the following conditions: having a glycated hemoglobin (HbA1c) level that is 6.5% or above; possessing a fasting plasma glucose (FPG) level of 7.0 mmol/L or more; having a random blood glucose level that goes beyond 11.1 mmol/L after undergoing a two‐hour oral glucose tolerance test (OGTT); presenting a random blood glucose reading of 11.1 mmol/L or higher; reporting through self‐reported information that they had previously been diagnosed with diabetes by a physician; or currently using insulin or other antihyperglycemic drugs to manage their diabetes.

### 2.3. Definition of Depression

Depression was measured using the PHQ‐9 questionnaire. The PHQ‐9 has 9 questions about common depression symptoms like feeling down, trouble sleeping, low energy, and appetite changes over the past 2 weeks [20]. Each question is scored from 0 (“never”) to 3 (“almost daily”), with total scores ranging from 0 to 27. In this study, scores of 10 or higher were considered clinically meaningful depression [21–23]. This cutoff helps identify people with moderate to severe depression.

### 2.4. Definition of TyG‐BMI

TyG‐BMI was calculated using the formula: ln(triglycerides (TG) [mg/dL] × FPG [mg/dL]/2) × BMI (kg/m^2^). The measurement of TG was carried out via enzymatic assays on a Roche Modular *p* analyzer, and the determination of FPG levels was performed using a hexokinase‐mediated enzymatic method on a Roche/Hitachi Cobas C 501 analyzer.

### 2.5. Covariates

The covariates considered in the study included age, gender (male or female), race (including Mexican American, other Hispanic, non‐Hispanic white, non‐Hispanic black, and other race), education level (grouped as below high school, high school, and above high school), marital status (categorized as married or living with a partner, widowed/divorced/separated, or never married), family PIR (with ranges of ≤ 1.30, 1.31–3.50, and > 3.50), BMI, smoking status (classified as never smoker, former smoker, or current smoker), drinking status (yes or no), hypertension (yes or no), CVD (yes or no), arthritis (yes or no), as well as biochemical indicators such as total cholesterol (TC), high‐density lipoprotein cholesterol (HDL‐C), and TG.

### 2.6. Statistical Analysis

In line with the NHANES analysis guidelines, all analyses took into account sample weights to reflect the complex survey design. This study is a retrospective cross‐sectional analysis of a publicly available dataset. Participants were divided into four groups based on the quartiles of the TyG‐BMI index: Q1: < 232.44, Q2: 232.44–270.05, Q3: 270.05–314.04, and Q4: > 314.04. Data are summarized as percentages (%) for categorical variables and mean ± standard deviation (SD)/medians (interquartile range, IQR) for continuous variables. Group comparisons used ANOVA (normal data), Kruskal–Wallis (non‐normal), or chi‐square tests (categorical). Logistic regression models were used to assess the relationship between TyG‐BMI and depression, calculating odds ratios (OR) and 95% confidence intervals (CI). Three models were used in this analysis. Model 1 was unadjusted; Model 2 adjusted for age, gender, race, education level, marital status, family PIR, and BMI; and Model 3 further adjusted for drinking status, smoking status, hypertension, CVD, arthritis, TC, HDL‐C, and TG based on Model 2. Additionally, restricted cubic spline (RCS) models were applied to analyze the dose‐response relationship between TyG‐BMI and depression.

In order to determine if there were any potential variations in the relationship between TyG‐BMI and depression, subgroup analyses were performed, considering the following variables: gender (male or female), education level (grouped as below high school, high school, and above high school), family PIR (with ranges of ≤ 1.30, 1.31–3.50, and > 3.50), BMI, drinking status (yes or no), hypertension (yes or no), CVD (yes or no), and arthritis (yes or no). We employed multivariable logistic models to assess subgroup variations, with interaction effects verified through likelihood ratio testing.

All statistical analyses were conducted using *R* software (version 4.3.3; *R* Foundation for Statistical Computing, Vienna, Austria). The threshold for statistical significance was established at a two‐sided *p* value < 0.05.

## 3. Results

### 3.1. Baseline Characteristics

Table 1 presents the baseline characteristics of study participants stratified by TyG‐BMI quartiles. The study cohort comprised 2584 participants with a mean age of 73.29 ± 5.52 years, of whom 1412 (54.64%) were male. Compared to lower TyG‐BMI quartiles, participants in the highest TyG‐BMI quartile exhibited distinct characteristics: younger mean age, higher proportion of females, greater representation of non‐Hispanic White, elevated BMI levels, increased prevalence of current smokers, and higher rates of hypertension, CVD, and arthritis, along with decreased HDL‐C and elevated TG levels.

**Table 1:** Characteristics of study participants by TyG‐BMI quartile.

Variable	Total (*N* = 2584)	Quartiles of TyG‐BMI				*p* value
		Q1: < 232.44 (*N* = 646)	Q2: 232.44–270.05 (*N* = 647)	Q3: 270.05–314.04 (*N* = 645)	Q4: > 314.04 (*N* = 646)	
Age (years), Mean ± SD	73.29 ± 5.52	74.65 ± 5.92	73.82 ± 5.52	72.80 ± 5.32	71.88 ± 4.87	< 0.001
Gender, *n* (%)						< 0.001
Male	1412 (54.64)	373 (57.74)	377 (58.27)	353 (54.73)	309 (47.83)	
Female	1172 (45.36)	273 (42.26)	270 (41.73)	292 (45.27)	337 (52.17)	
Race, *n* (%)						< 0.001
Mexican American	331 (12.81)	64 (9.91)	84 (12.98)	91 (14.11)	92 (14.24)	
Other Hispanic	219 (8.48)	52 (8.05)	64 (9.89)	52 (8.06)	51 (7.89)	
Non‐Hispanic White	1301 (50.35)	333 (51.55)	309 (47.76)	315 (48.84)	344 (53.25)	
Non‐Hispanic Black	545 (21.08)	121 (18.73)	135 (20.87)	152 (23.57)	137 (21.21)	
Other Race	188 (7.28)	76 (11.76)	55 (8.50)	35 (5.42)	22 (3.41)	
Educational level, *n* (%)						0.427
Below high school	970 (37.54)	239 (37.00)	250 (38.64)	234 (36.28)	247 (38.24)	
High school	652 (25.23)	171 (26.47)	167 (25.81)	147 (22.79)	167 (25.85)	
Above high school	962 (37.23)	236 (36.53)	230 (35.55)	264 (40.93)	232 (35.91)	
Marital status, *n* (%)						0.104
Married/Living with partner	1475 (57.08)	372 (57.59)	386 (59.66)	364 (56.43)	353 (54.64)	
Widowed/Divorced/Separated	1022 (39.55)	256 (39.63)	248 (38.33)	256 (39.69)	262 (40.56)	
Never married	87 (3.37)	18 (2.78)	13 (2.01)	25 (3.88)	31 (4.80)	
Family PIR, *n* (%)						0.285
≤ 1.3	846 (32.74)	201 (31.11)	202 (31.22)	212 (32.87)	231 (35.76)	
1.3–3.5	1180 (45.67)	296 (45.82)	291 (44.98)	303 (46.98)	290 (44.89)	
> 3.5	558 (21.59)	149 (23.07)	154 (23.80)	130 (20.15)	125 (19.35)	
BMI (kg/m2), Mean ± SD	30.38 ± 6.42	23.79 ± 2.40	27.97 ± 2.07	31.26 ± 2.38	38.49 ± 5.79	< 0.001
Smoking status, *n* (%)						< 0.001
Former smoker	216 (8.36)	85 (13.16)	47 (7.27)	51 (7.91)	33 (5.11)	
Never smoker	1190 (46.05)	302 (46.75)	310 (47.91)	284 (44.03)	294 (45.51)	
Current smoker	1178 (45.59)	259 (40.09)	290 (44.82)	310 (48.06)	319 (49.38)	
Drinking status, *n* (%)						0.128
No	922 (35.68)	217 (33.59)	236 (36.48)	217 (33.64)	252 (39.01)	
Yes	1662 (64.32)	429 (66.41)	411 (63.52)	428 (66.36)	394 (60.99)	
Hypertension, *n* (%)						< 0.001
No	574 (22.21)	200 (30.96)	157 (24.27)	120 (18.60)	97 (15.02)	
Yes	2010 (77.79)	446 (69.04)	490 (75.73)	525 (81.40)	549 (84.98)	
CVD, *n* (%)						< 0.001
No	1657 (64.13)	440 (68.11)	416 (64.30)	430 (66.67)	371 (57.43)	
Yes	927 (35.87)	206 (31.89)	231 (35.70)	215 (33.33)	275 (42.57)	
Arthritis, *n* (%)						< 0.001
No	1196 (46.28)	353 (54.64)	319 (49.3)	297 (46.05)	227 (35.14)	
Yes	1388 (53.72)	293 (45.36)	328 (50.7)	348 (53.95)	419 (64.86)	
TC (mmol/L), Mean ± SD	4.62 ± 1.15	4.63 ± 1.13	4.56 ± 1.11	4.65 ± 1.26	4.64 ± 1.09	0.464
HDL‐C (mmol/L), Mean ± SD	1.31 ± 0.41	1.53 ± 0.48	1.33 ± 0.39	1.22 ± 0.34	1.18 ± 0.32	< 0.001
TG (mg/dL), Median (IQR)	137.00 (94.00, 202.00)	96.00 (69.00, 131.00)	127.00 (93.00, 180.00)	162.00 (113.00, 229.00)	179.50 (128.00, 259.00)	< 0.001

*Note:* TyG‐BMI: triglyceride‐glucose‐body mass index, PIR: poverty‐to‐income ratio, CVD: cardiovascular diseases, HDL‐C: high‐density lipoprotein cholesterol, TG: triglycerides, IQR: interquartile range.

Abbreviations: BMI = body mass index, SD = standard deviation, TC = total cholesterol.

### 3.2. The Association of TyG‐BMI With Depression

The univariate analysis showed that gender, educational level, marital status, family PIR, BMI, CVD, arthritis, TC, TG, and TyG‐BMI were associated with depression (Table 2). Notably, in the baseline characteristic comparisons (Table 1), no statistically significant associations were found between TyG‐BMI and educational level, marital status, family PIR, drinking status, or TC levels (all *p* > 0.05). In unadjusted analysis, participants in the highest TyG‐BMI quartile (Q4: > 314.04) demonstrated more than a 2.5‐fold increased risk of depression compared to the lowest quartile (Q1: < 232.44) (OR = 2.780, 95% CI: 1.826–4.232; *p* < 0.001). After full adjustment for potential confounders, this association remained significant, with an adjusted OR of 2.972 (95% CI: 1.264–6.988, *p* = 0.013) for Q4 versus Q1 (Table 3).

**TABLE 2:** Univariate logistic regression analysis of potential risk factors for depression.

Variable	OR (95%CI)	*p* value
Age	0.979 (0.949–1.009)	0.167

*Gender*		
Male	1.000 (reference)	
Female	1.811 (1.295–2.532)	< 0.001

*Race*		
Mexican American	1.000 (reference)	
Other Hispanic	1.273 (0.687–2.356)	0.439
Non‐Hispanic White	0.760 (0.486–1.190)	0.228
Non‐Hispanic Black	0.666 (0.394–1.128)	0.129
Other Race	0.708 (0.354–1.415)	0.325

*Educational level*		
Below high school	1.000 (reference)	
High school	0.798 (0.529–1.205)	0.281
Above high school	0.477 (0.317–0.717)	< 0.001

*Marital status*		
Married/Living with partner	1.000 (reference)	
Widowed/Divorced/Separated	1.629 (1.151–2.305)	0.006
Never married	2.599 (0.857–7.880)	0.091

*Family PIR*		
≤ 1.3	1.000 (reference)	
1.3–3.5	0.607 (0.427–0.864)	0.006
> 3.5	0.182 (0.099–0.331)	< 0.001
BMI	1.051 (1.030–1.073)	< 0.001

*Smoking status*		
Former smoker	1.000 (reference)	
Never smoker	0.647 (0.366–1.143)	0.132
Current smoker	0.836 (0.489–1.428)	0.508

*Drinking status*		
No	1.000 (reference)	
Yes	0.819 (0.584–1.149)	0.245

*Hypertension*		
No	1.000 (reference)	
Yes	1.418 (0.938–2.144)	0.097

*CVD*		
No	1.000 (reference)	
Yes	2.080 (1.478–2.927)	< 0.001

*Arthritis*		
No	1.000 (reference)	
Yes	3.262 (2.292–4.642)	< 0.001
TC	1.183 (1.004–1.394)	0.045
HDL‐C	0.853 (0.628–1.159)	0.307
TG	1.001 (1.000–1.003)	0.022
TyG‐BMI	1.005 (1.003–1.007)	< 0.001

*Note:* PIR poverty‐to‐income ratio, CVD cardiovascular diseases, HDL‐C high‐density lipoprotein cholesterol, TG triglycerides, TyG‐BMI, triglyceride‐glucose‐body mass index.

Abbreviations: BMI = body mass index, CI = confidence intervals, OR = odds ratio, TC = total cholesterol.

**TABLE 3:** Association between TyG‐BMI and depression.

Exposure	OR (95%CI)					
	Model 1	*p* value	Model 2	*p* value	Model 3	*p* value
Continuous	1.005 (1.003–1.007)	< 0.001	1.010 (1.003–1.016)	0.005	1.015 (1.000–1.030)	0.043

*Quartiles of TyG-BMI*						
Q1: < 232.44	Reference		Reference		Reference	
Q2: 232.44–270.05	1.004 (0.620–1.626)	0.987	1.072 (0.640–1.799)	0.789	1.047 (0.596–1.840)	0.870
Q3: 270.05–314.04	1.468 (0.934–2.306)	0.095	1.581 (0.960–2.605)	0.072	1.582 (0.910–2.752)	0.103
Q4: > 314.04	2.780 (1.826–4.232)	< 0.001	3.152 (1.457–6.820)	0.004	2.972 (1.264–6.988)	0.013
*p* for trend	< 0.001		0.003		0.007	

*Note:* Model 1 adjust for: none. Model 2 adjust for: age, gender, race, education level, marital status, family PIR, and BMI. Model 3 adjust for: age, gender, race, education level, marital status, family PIR, BMI, drinking status, smoking status, hypertension, CVD, arthritis, TC, HDL‐C, and TG. TyG‐BMI, triglyceride‐glucose‐body mass index.

Abbreviations: CI = confidence intervals, OR = odds ratio.

**FIGURE 2:**
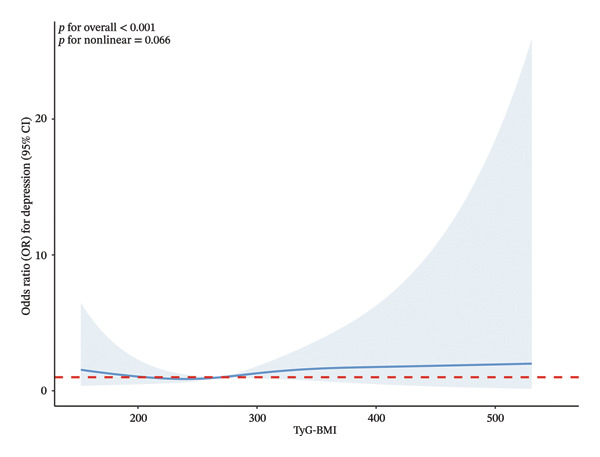
Dose‐response relationship between TyG‐BMI and the risk of depression. The solid line represents the adjusted odds ratio (OR), and the shaded area indicates the 95% confidence interval (95% CI). The analysis was performed using a restricted cubic spline model with four knots, adjusted for all covariates in Model 3 (age, gender, race, education level, marital status, family PIR, BMI, drinking status, smoking status, hypertension, CVD, arthritis, TC, HDL‐C, and TG).

As illustrated in Figure 2, we constructed an RCS model to flexibly characterize and visually present the dose‐response relationship between TyG‐BMI and depression risk. After comprehensive adjustment for covariates in Model 3, the analytical results revealed a statistically significant linear association between elevated TyG‐BMI levels and increased depression risk (*p* for overall < 0.001; *p* for nonlinearity = 0.066).

### 3.3. Subgroup Analysis

To further investigate potential variations in the association between TyG‐BMI and depression across different populations, stratified analyses were conducted based on gender, educational level, family PIR, drinking status, and disease status (including hypertension, CVD, and arthritis). The subgroup analyses revealed no statistically significant interaction effects between TyG‐BMI and any of the stratification variables (Table 4).

**TABLE 4:** Association between TyG‐BMI and the risk of depression in various subgroups.

	OR (95%CI)				*p* for interaction
	Q1: < 232.44	Q2: 232.44–270.05	Q3: 270.05–314.04	Q4: > 314.04	
Gender					0.485
Male	Reference	0.941 (0.501–1.778)	0.816 (0.376–1.791)	1.401 (0.499–4.026)	
Female	Reference	0.759 (0.417–1.383)	0.809 (0.418–1.584)	0.784 (0.323–1.930)	
Educational level					0.940
Below high school	Reference	0.711 (0.383–1.320)	0.756 (0.359–1.600)	0.762 (0.268–2.157)	
High school	Reference	1.118 (0.449–2.827)	0.870 (0.274–2.805)	1.797 (0.427–7.997)	
Above high school	Reference	0.952 (0.406–2.255)	0.958 (0.391–2.430)	1.113 (0.329–3.863)	
Family PIR					0.412
≤ 1.3	Reference	0.695 (0.373–1.292)	0.664 (0.324–1.362)	0.530 (0.200–1.398)	
1.3–3.5	Reference	0.910 (0.462–1.800)	1.009 (0.468–2.226)	1.396 (0.485–4.134)	
> 3.5	Reference	2.137 (0.456–11.263)	2.289 (0.331–18.028)	9.251 (0.895–127.820)	
Drinking status					0.129
No	Reference	0.753 (0.378–1.505)	0.702 (0.304–1.643)	0.621 (0.192–2.024)	
Yes	Reference	0.853 (0.485–1.499)	0.882 (0.472–1.667)	1.295 (0.572–2.999)	
Hypertension					0.470
No	Reference	1.242 (0.481–3.282)	0.841 (0.210–3.413)	1.626 (0.245–11.003)	
Yes	Reference	0.799 (0.485–1.317)	0.884 (0.512–1.542)	1.018 (0.495–2.123)	
CVD					0.073
No	Reference	0.946 (0.520–1.732)	0.572 (0.277–1.176)	0.655 (0.243–1.727)	
Yes	Reference	0.767 (0.405–1.451)	1.354 (0.657–2.855)	1.617 (0.611–4.405)	
Arthritis					0.447
No	Reference	0.537 (0.236–1.203)	0.757 (0.303–1.954)	0.697 (0.180–2.774)	
Yes	Reference	1.061 (0.630–1.798)	0.889 (0.486–1.642)	1.212 (0.553–2.708)	

*Note:* Stratified analyses were performed based on Model 3, wherein the models were not adjusted for the variables used for stratification themselves. PIR, poverty‐to‐income ratio, CVD, cardiovascular diseases.

Abbreviations: CI = confidence intervals, OR = odds ratio.

## 4. Discussion

This study provides novel evidence that elevated TyG‐BMI is independently associated with increased depression risk in elderly patients with diabetes, even after adjusting for sociodemographic and metabolic confounders. The dose‐response analysis revealed a linear relationship, suggesting a potential threshold effect of TyG‐BMI on depressive symptoms. These findings align with previous reports linking IR to depression but extend current knowledge by demonstrating the superiority of TyG‐BMI—a composite index integrating lipid, glucose, and obesity parameters—over traditional IR markers in predicting psychological comorbidity. The consistency of the TyG‐BMI–depression association across all stratified subgroups—including those defined by gender, socioeconomic status, and key comorbidities—suggests that this relationship is a pervasive feature in the elderly diabetic population and is not meaningfully modified by these factors.

The strong association between elevated TyG‐BMI and depression risk underscores its value as a composite biomarker integrating lipid‐glucose metabolism and obesity [24]. Unlike traditional IR markers (e.g., HOMA‐IR), TyG‐BMI does not require insulin measurement, enhancing its clinical accessibility for mental‐metabolic comorbidity screening [25]. Our findings align with prior studies showing TyG‐BMI’s superior predictive capacity for diabetes complications and its correlation with neuroinflammation pathways implicated in depression [26–28]. This suggests that TyG‐BMI may capture multifaceted metabolic disturbances that synergistically contribute to psychiatric vulnerability.

The observed linear dose‐response relationship supports the hypothesis that chronic low‐grade inflammation and oxidative stress—hallmarks of IR—mediate the TyG‐BMI–depression association [29]. IR‐driven dysregulation of the HPA axis and serotonin metabolism may directly impair mood regulation [30]. Additionally, visceral adiposity, reflected in elevated BMI, secretes pro‐inflammatory cytokines (e.g., IL‐6 and TNF‐α) that disrupt blood‐brain barrier integrity and promote neuroinflammation, further exacerbating depressive symptoms [31]. These mechanisms are particularly relevant in elderly diabetics, who often exhibit accelerated metabolic aging.

The lack of significant subgroup interactions implies that TyG‐BMI’s predictive utility is consistent across diverse sociodemographic and clinical profiles. This stability strengthens its potential as a universal screening tool for depression in elderly diabetics, a population with high metabolic‐psychiatric comorbidity burdens [32, 33]. However, the “obesity paradox” observed in some geriatric studies—where higher BMI correlates with lower mortality—warrants caution in interpreting BMI‐related indices, necessitating age‐specific cutoff values for TyG‐BMI [34].

Prospective cohort studies are needed to clarify whether TyG‐BMI elevation precedes depression onset or vice versa. Additionally, interventional trials could test whether improving TyG‐BMI through lifestyle modifications (e.g., Mediterranean diet, aerobic exercise) reduces depression risk. Further mechanistic research should explore tissue‐specific insulin signaling (e.g., brain vs. peripheral tissues) and genetic/epigenetic factors modulating the TyG‐BMI–depression axis.

This study has several limitations. The cross‐sectional design of this study precludes causal inference between TyG‐BMI and depressive symptoms, while potential measurement biases may exist due to self‐reported depression assessments via the PHQ‐9, particularly in older adults with possible cognitive impairments. Furthermore, unmeasured confounding factors, such as antidepressant medication use, dietary patterns, and physical activity levels, might have influenced the observed associations.

## 5. Conclusion

TyG‐BMI serves as a reliable predictor of depression risk in elderly individuals with diabetes, highlighting its dual role in reflecting metabolic dysfunction and mental health vulnerability. Integrating TyG‐BMI into routine clinical assessments may facilitate early identification of high‐risk populations for targeted interventions. Future longitudinal studies are warranted to elucidate causal mechanisms and validate its utility in depression prevention strategies.

**Nomenclature**
ADA: American Diabetes Association
ANOVA: Analysis of variance
BMI: Body mass index
CI: Confidence interval
CVD: Cardiovascular disease
FPG: Fasting plasma glucose
GBD: Global Burden of Disease
HbA1c: Hemoglobin A1c
HDL‐C: High‐density lipoprotein cholesterol
HOMA‐IR: Homeostatic model assessment of insulin resistance
HPA: Hypothalamic‐pituitary‐adrenal
IL‐6: Interleukin‐6
IR: Insulin resistance
IRB: Institutional Review Board
NHANES: National Health and Nutrition Examination Survey
OGTT: Oral glucose tolerance test
OR: Odds ratio
PHQ‐9: Patient Health Questionnaire‐9
PIR: Poverty‐to‐income ratio
RCS: Restricted cubic spline
SD: Standard deviation
TC: Total cholesterol
TG: Triglycerides
TNF‐α: Tumor necrosis factor‐alpha
TyG‐BMI: Triglyceride‐glucose‐body mass index

## Author Contributions

Hong Qian and Dean Wu designed the research. Hong Qian, Shanglin Song, and Zhengyu Zhang collected and organized data. Hong Qian, Shanglin Song, and Yuan Yuan analyzed the data. Hong Qian and Dean Wu drafted the manuscript, and Dean Wu contributed to the critical revision of the manuscript.

## Funding

This study was funded by Soft Science Project of Gansu Provincial Department of Science and Technology (22JR11RA087).

## Disclosure

All authors contributed to the manuscript and approved the submitted version.

## Ethics Statement

The US National Health and Nutrition Examination Survey (NHANES) protocol was approved by both the NHANES Institutional Review Board (IRB) and the National Center for Health Statistics (NCHS) Research Ethics Review Board. Written informed consent was obtained from all participants. Because this secondary analysis used publicly available de‐identified data, no additional IRB approval was required.

## Consent

Please see the Ethics Statement.

## Conflicts of Interest

The authors declare no conflicts of interest.

## Data Availability

The data used in this study are from a public database at https://wwwn.cdc.gov/nchs/nhanes, which can be accessed by everyone through the links provided in the paper.
